# Lipid Nanocarriers Overlaid with Chitosan for Brain Delivery of Berberine via the Nasal Route

**DOI:** 10.3390/ph15030281

**Published:** 2022-02-24

**Authors:** Hadel A. Abo El-Enin, Mohammed H. Elkomy, Ibrahim A. Naguib, Marwa F. Ahmed, Omar A. Alsaidan, Izzeddin Alsalahat, Mohammed M. Ghoneim, Hussein M. Eid

**Affiliations:** 1 Department of Pharmaceutics and Industrial Pharmacy, College of Pharmacy, Taif University, P.O. Box 11099, Taif 21944, Saudi Arabia; hadel.a@tu.edu.sa; 2 Department of Pharmaceutics, College of Pharmacy, Jouf University, P.O. Box 2014, Sakaka 72341, Saudi Arabia; osaidan@ju.edu.sa; 3 Department of Pharmaceutical Chemistry, College of Pharmacy, Taif University, P.O. Box 11099, Taif 21944, Saudi Arabia; marwa.farg@tu.edu.sa; 4 Complement Biology Group, Systems Immunity Research Institute, School of Medicine, Cardiff University, Cardiff CF14 4XW, UK; alsalahati@cardiff.ac.uk; 5 Department of Pharmaceutical Chemistry and Pharmacognosy, Faculty of Pharmacy, Applied Science Private University, Amman 11931, Jordan; 6 Department of Pharmacy Practice, Faculty of Pharmacy, AlMaarefa University, P.O. Box 71666, Ad Diriyah 13713, Saudi Arabia; mghoneim@mcst.edu.sa; 7 Department of Pharmaceutics and Industrial Pharmacy, Faculty of Pharmacy, Beni-Suef University, Beni-Suef 62521, Egypt; hussien.eid@pharm.bsu.edu.eg

**Keywords:** intranasal, lipidic nanoparticles, coatings, nutraceuticals, brain targeting, biodistribution

## Abstract

This research aimed to design, optimize, and evaluate berberine-laden nanostructured lipid carriers overlaid with chitosan (BER-CTS-NLCs) for efficient brain delivery via the intranasal route. The nanostructured lipid carriers containing berberine (BER-NLCs) were formulated via hot homogenization and ultrasonication strategy and optimized for the influence of a variety of causal variables, including the amount of glycerol monostearate (solid lipid), poloxamer 407 (surfactant) concentration, and oleic acid (liquid lipid) amount, on size of the particles, entrapment, and the total drug release after 24 h. The optimal BER-NLCs formulation was then coated with chitosan. Their diameter, in vitro release, surface charge, morphology, ex vivo permeability, pH, histological, and in vivo (pharmacokinetics and brain uptake) parameters were estimated. BER-CTS-NLCs had a size of 180.9 ± 4.3 nm, sustained-release properties, positive surface charge of 36.8 mV, and augmented ex-vivo permeation via nasal mucosa. The histopathological assessment revealed that the BER-CTS-NLCs system is safe for nasal delivery. Pharmacokinetic and brain accumulation experiments showed that animals treated intranasally with BER-CTS-NLCs had substantially greater drug levels in the brain. The ratios of BER brain/blood levels at 30 min, AUC_brain_/AUC_blood_, drug transport percentage, and drug targeting efficiency for BER-CTS-NLCs (IN) were higher compared to BER solution (IN), suggesting enhanced brain targeting. The optimized nanoparticulate system is speculated to be a successful approach for boosting the effect of BER in treating CNS diseases, such as Alzheimer’s disease, through intranasal therapy.

## 1. Introduction

Natural products have recently acquired much attention because of their anti-inflammatory, antiplatelet, antioxidant, and other health benefits [1,2]. For decades, the isoquinoline alkaloid berberine (BER) has been used to treat dementia and other mental disorders [3]. Recent investigations have shown that BER is effective against Alzheimer’s disease (AD), among other psychological and neurological-based disorders [4,5,6]. AD is a chronic neurological condition that worsens with time. AD is a major challenge worldwide [7]. Around 25% of AD occurs in familial communities, which are marked by big family units, high rates of consanguineous marriages, and high birth rates, making hereditary disorders a major health issue [8]. This deadly disease is characterized by cognitive impairment, impairment of fundamental functions, behavioral abnormalities, and a range of neuropsychiatric symptoms [9,10].

Individuals with AD are now treated with medications that raise acetylcholine levels in brain tissue, such as rivastigmine, galantamine, and donepezil [11]. However, none of these medicines completely alleviates cognitive impairment and cholinergic system insufficiency in AD patients [12]. As a result, the current focus has turned to identifying new moieties for managing and treating AD [13,14]; one of these moieties is BER. The amelioration of A1–40-induced cognitive impairments, as well as the inhibition of indoleamine 2, 3-dioxygenase, and acetyl/butyl cholinesterases, are the primary mechanisms via which BER exerts its anti-AD actions [15,16,17]. On the other hand, poor systemic bioavailability (<5%), limited CNS penetration, and diminished intestinal absorption of BER all work against these positive effects [18]. As a consequence, new routes and nanoparticulate delivery systems for CNS delivery of this nutraceutical are critical to explore.

Drug delivery systems based on nanotechnology have emerged as potential vehicles for encapsulating, protecting, as well as delivering embedded pharmaceuticals to the desired site at a specified rate and/or extent. Novel delivery systems have been devised to improve BER’s physicochemical characteristics and brain bioavailability. Carbon nanotubes [19], chitosan nanoparticles [20], and polymeric nanoparticles [21] formulations of BER have been reported.

Drugs may be transported to the brain through the nose without crossing the BBB, lowering plasma levels and decreasing side effects [22]. The olfactory and/or trigeminal nerve pathways, which run along the top of the nose cavity, are most likely facilitating the nasal route. They facilitate the entry of medicines into the olfactory bulb and brainstem, respectively [23]. Interestingly, this route is comfortable, safe, non-invasive, and feasible.

Numerous approaches for brain delivery have been investigated, with nano-systems emerging as the most promising. Nanostructured lipid carriers (NLCs), a new generation of lipid nanoparticles, were designed to address some of the drawbacks of solid lipid nanoparticles (SLNs). NLCs include both solid and liquid lipid matrix [24]; this results in a matrix with a lower ordered structure containing several imperfections, which boosts drug entrapment and decreases drug leakage [25]. Due to their lipophilicity, NLCs have been utilized to deliver bioactive constituents to the brain [26]. They retain their low toxicity and high tolerance due to their composition of biodegradable lipids [27]. Numerous studies have utilized NLCs for nose-to-brain delivery of several drugs such as ketoconazole [28], rivastigmine [29], and clozapine [30]. Since the delivery route should be paved to precisely target BER to the brain in order to maximize impact and minimize potential adverse effects, encapsulating BER in NLCs with intranasal administration seems to be the perfect combination.

However, the duration of stay of nanoparticles in the nasal cavity is significantly short owing to mucociliary clearance, which results in insufficient drug absorption. Chitosan (CTS), a naturally occurring polymer, has recently been used in nasal delivery systems due to its mucoadhesion merit, which boosts drug penetration through the nasal mucosa and impedes mucociliary clearance as well [31]. Additionally, CTS is of low toxicity and high biocompatibility.

Fabrication and evaluation of BER-loaded NLCs for the management of AD through oral administration have been described elsewhere [32]. This study aims to evaluate the effectiveness of nanostructured lipid carriers overlaid with chitosan in improving BER brain transport via the intranasal route. As far as we know, our investigation is the first to explore the merits of NLCs coated with CTS as a delivery system for BER through intranasal administration. The physical and morphological properties of BER-CTS-NLCs were tailored, optimized, and scrutinized to be suitable for the nose to brain delivery. Moreover, upon intranasal delivery, the pharmacokinetic characteristics and effectiveness of BER-CTS-NLCs for brain targeting were investigated.

## 2. Results and Discussion

### 2.1. Formulation Considerations

It is essential to select suitable components during the formulation of NLCs in order to obtain optimal uniform PS, stability, and entrapment. Surfactants, for instance, significantly affect the quality and PS of NLCs. Poloxamer 407 was used in this study owing to its resistance to particle agglomeration and suppression of P-glycoproteins involved in the brain endothelial cell efflux mechanism [33]. By expelling drug molecules from cells, this type of P-glycoproteins prevents the buildup of specific drug molecules in the brain. It has been proposed that inhibiting P-glycoproteins improves drug transport into the brain and hence the bioavailability of brain-targeted medicines [34]. Additionally, selecting an appropriate solid lipid for the system is critical, as it represents the matrix required for drug loading and affects the amount of drug solubilized and hence entrapped. Singh et al. reported that intranasal administration of NLCs containing GMS in conjunction with OA resulted in high drug levels in the brain [35].

### 2.2. Design, Statistical Analysis, and Optimization

#### 2.2.1. Particle Diameter, Entrapment, Surface Charge, and Cumulative Drug Release at 24 h

**Figure 1:**
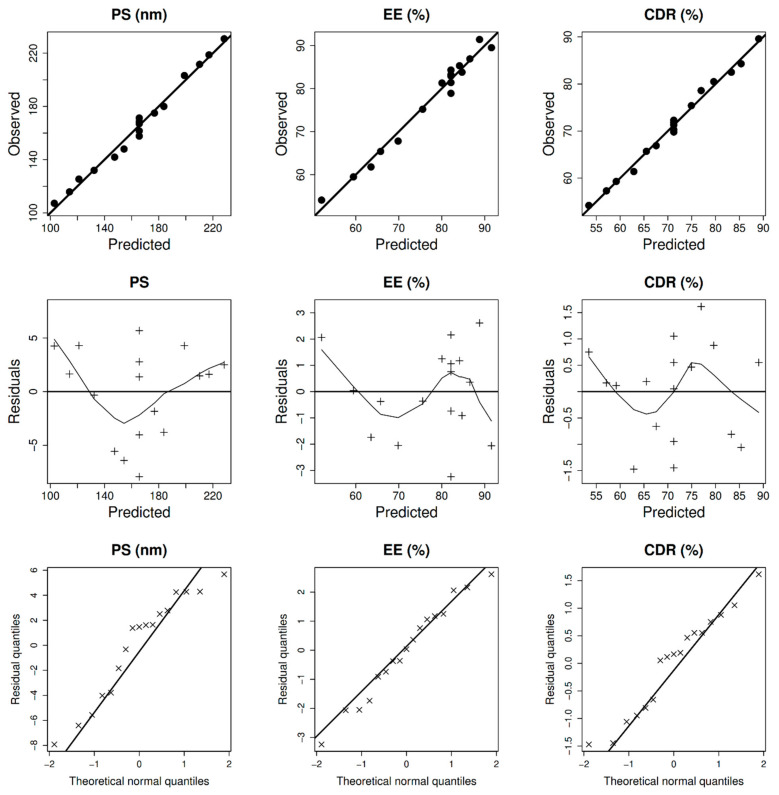
Model diagnostics plots of BER-NLCs size (PS), entrapment (EE%), and cumulative drug released (CDR%).

Table 1 displays the PS, EE, ZP, and CDR results of seventeen BER-NLCs formulations with different GMS amounts, poloxamer 407 concentration, and OA amounts. The average PS, EE, and CDR ranged between 107.2–230.8 nm, 54.1–91.4%, and 54.2–89.6%, respectively. The broad range of response values suggests that changes in GMS, poloxamer 407 concentrations, and OA, all or in part, may have a significant impact on NLCs characteristics. ANOVA Type III was used to recognize the precise causes of PS, EE, and CDR variability. All formulation variables were shown to be significant predictors of PS, EE, and CDR (*p*-value < 0.05). The relations between the independent variables: GMS amount (X_1_), poloxamer 407 concentration (X_2_) and OA amount (X_3_), and the response variables ((Y1: PS), (Y2: EE %), and (Y3: CDR %)) was best described by the mathematical equations mentioned in Table 2. The models effectively described the observed variability, as shown by the models’ insignificant lack of fit error (Table 2). The diagnostic model plots in Figure 1 demonstrate that the models properly described the data without apparent residual errors that followed a normal distribution.

ZP values of the NLC formulations ranged between −35.1 and −27.5 mV (Table 1). The high negative values mean that the formed nanoparticles are expected to be stable based on ZP results [36]. ANOVA Type III analysis of ZP values revealed no significant impacts of GMS, OA, or poloxamer 407. Therefore, ZP was excluded from the optimization step.

#### 2.2.2. Optimization of Size

**Figure 2:**
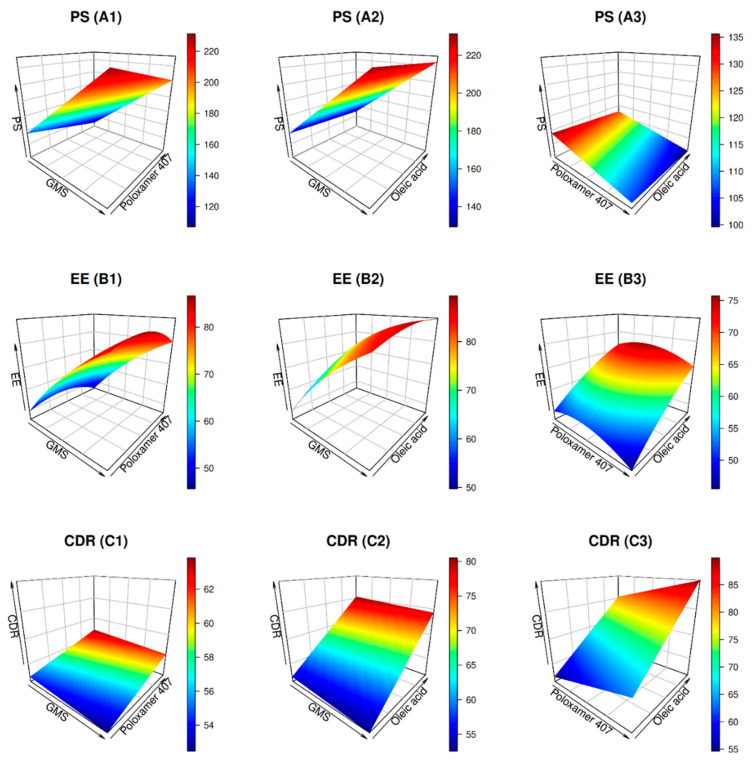
3D plots for the effects of GMS amount (mg), poloxamer 407 concentration (% *w*/*w*), and oleic acid amount (mg) on BER-CTS-NLCs size (PS), entrapment (EE%), and cumulative drug released (CDR%).

As shown in Figure 2 (A1), raising the GMS concentration from 100 to 200 mg increased the PS from 133 to more than 230 nm. The effect of GMS on PS is as follows: raising solid lipid concentration increased the viscosity of the dispersed phase, leading to particle agglomeration and subsequent increase in diameter, and vice versa [37]. When poloxamer 407 concentration was raised from 1 to 2%, the PS dropped from 133 to 110 nm (Figure 2 (A1, A3)). The negative correlation between poloxamer 407 and PS is due to decreased interfacial tension between the ambient phase and the lipid, which results in particle partitioning [38]. Additionally, the increase in OA concentration from 15 to 30 mg was followed by a slight reduction in PS from 123 to 117 nm (Figure 2 (A2, A3)). This is because the liquid lipid changes the viscosity of the solid matrix, causing smaller particles to form [39].

#### 2.2.3. Optimization of Entrapment

Entrapment of more than 90% was obtained when the GMS amount was more than 190 mg (Figure 2 (B1, B2)), poloxamer 407 less than 1.5% (Figure 2 (B1, B3)), and OA above 25 (Figure 2 (B2, B3)). Increasing the amount of solid and liquid lipids, thus decreasing poloxamer 407 concentrations to a certain extent, increases the EE. It was found that increasing the concentration of the GMS produced an increase in drug EE. This may be attributable to BER’s hydrophobic behavior and partitioning into the internal lipid phase with increased solid lipid and reduced poloxamer 407 [40]. According to Ferreira and colleagues, surfactants permitted drugs to be partitioned from the nanoparticle’s internal phase to the external phase [41]. As a result, the entrapment of the drug is diminished. Liquid lipids create different crystal defects in solid lipids and produce flaws in highly ordered crystals, which explains why OA benefits EE. As a result, this space becomes more tightly packed with drug molecules [42].

#### 2.2.4. Optimization of CDR

The formulations with low GMS levels and high levels of OA and poloxamer 407 had the greatest CDR, as illustrated in Figure 2 (C1–C3). Formulations with high levels of GMS and low levels of OA and poloxamer 407, on the other hand, exhibited the lowest CDR. PS and EE are both parameters that affect drug release from a certain carrier system; hence in vitro drug release patterns were predicted to differ. Consequently, the formulations with the lowest PS and the highest EE exhibited the largest CDR over time. As shown in Figure 2 (C2, C3), OA substantially affected the CDR. It is noteworthy that the PS dropped when OA levels were increased, increasing specific surface area and, consequently, CDR.

#### 2.2.5. Composition of Optimal BER-NLCs

The optimization conditions for the NLCs formulation were set to minimize the particle size and maximize the entrapment efficiency and cumulative drug release after 24 h. Table 3 presents the optimal combination of formulation parameters as determined by the Design Expert^®^ program and desirability algorithm (0.84). The optimal NLCs are characterized by low GMS amount and high OA amount, and poloxamer 407 concentration. This combination yielded 142.1 ± 5.7 nm particles capable of holding 80.3 ± 3.1% of the added drug and releasing 85.6 ± 4.2% after 24 h. These measured values were very similar to the values predicted by the model, 119.8 nm, 76.1%, and 88.2, respectively, proving the accuracy of the optimization methods used in this analysis (Table 3).

### 2.3. Characterization of BER-CTS-NLCs

#### 2.3.1. BER-CTS-NLCs Size, Surface Charge, and Morphological Evaluation

As anticipated, CTS coated NLCs exhibited a much higher PS (180.9 ± 4.3) than uncoated NLCs (142.1 ± 5.7), suggesting that CTS attached effectively to the NLCs’ surface, resulting in their increased size [43]. The surface charge of the particles is represented by ZP, which reflects the extent of repulsion between similarly charged particles in the dispersion, preventing particle aggregation and demonstrating the stability of the dispersion. According to the Derjaguine Landaue Verweye Overbeek (DLVO) theory, if the surface charge is higher than 30 mV (negative or positive), the system is assumed to be stable [36]. The high positive charge of BER-CTS-NLCs (36.8 mV) therefore suggests their good stability. This high stability may be due to steric stabilization of the non-ionic surfactant (Poloxamer 407) [44].

**Figure 3:**
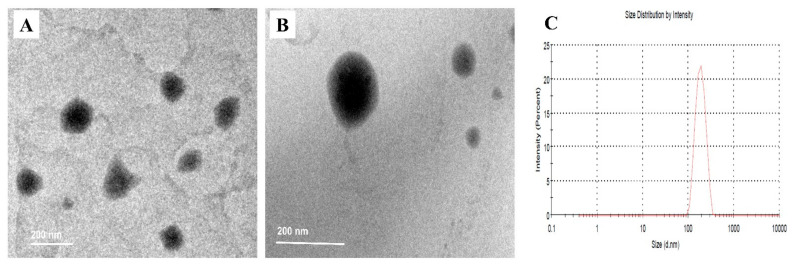
TEM morphology of (**A**) BER-NLCs, (**B**) BER-CTS-NLCs, and (**C**) particle size and PDI of BER-CTS-NLCs.

Figure 3 illustrates the TEM ultrastructure of BER-NLCs and BER-CTS-NLCs. The BER-NLCs micrograph showed a spherical shape due to the nano-droplets (Figure 3A). The micrographic photo presentation of the BER-CTS-NLCs dispersion was noticeably different (Figure 3B); it detected a spherical-shaped particle with a prominent double-layer structure. The presence of CTS polymer evenly surrounding the surface of NLC particles resulted in slightly larger particles, which was also consistent with the PS analysis experimental findings (Figure 3C).

#### 2.3.2. In Vitro Release Studies of BER

**Figure 4:**
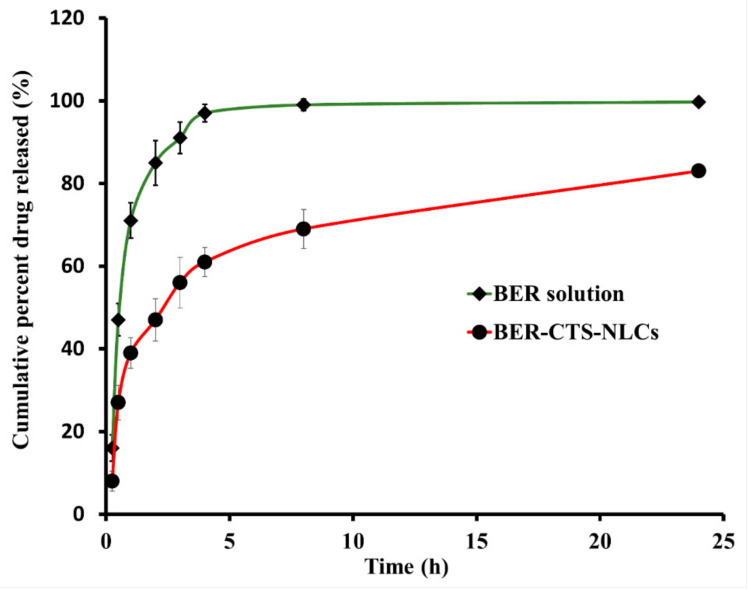
In-vitro release profiles of BER from BER-CTS-NLCs and BER solution.

The in vitro release of BER-CTS-NLCs vs. BER-SOL in SNES is shown in Figure 4. The cumulative quantity of BER released from BER-CTS-NLCs and BER-SOL was plotted against time. The BER-SOL exhibited fast BER release, with more than 95% cumulative release within 4 h. Conversely, the release of BER from BER-CTS-NLCs was 61.7% within the same time range. Figure 4 depicts a biphasic drug release pattern from BER-CTS-NLCs, with a burst release occurring within the first 60 min (39.2%) and a gradual release over 24 h.

#### 2.3.3. Ex Vivo BER Permeation Studies

**Figure 5:**
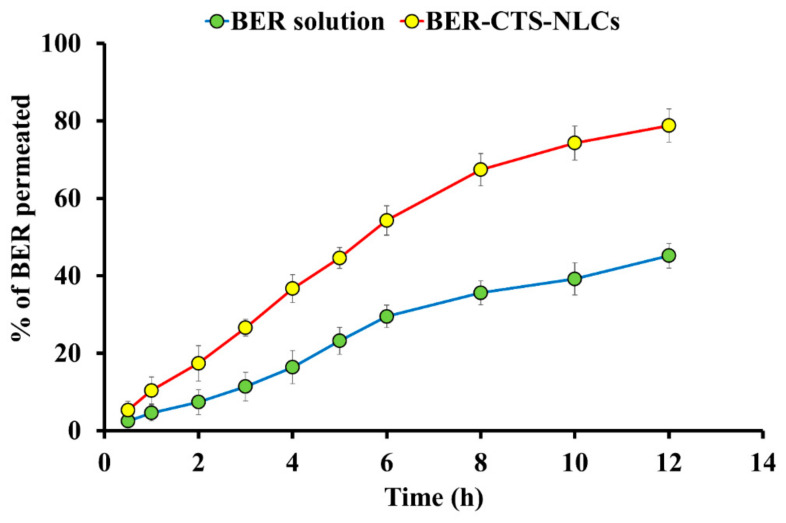
Ex vivo permeation profiles of BER from BER-CTS-NLCs and BER solution.

Sheep nasal epithelium was selected for permeation analysis because of similar histology to human nasal epithelium [45]. NLCs have played a crucial role in enhancing lipophilic drug molecules’ permeability. Within 12 h, 78.8 ± 4.3% (472.8 ± 25.87 µg/cm^2^) of BER was permeated from BER-CTS-NLCs, while 45.2 ± 3.2% (271.2 ± 19.25 µg/cm^2^) of BER was permeated from the BER-SOL. The findings of this experiment indicate that BER-CTS-NLCs permeated more than BER-SOL (*p* < 0.05) (Figure 5 and Table 4). The permeability coefficient (Papp) and steady-state flux (Jss) of BER-CTS-NLCs and BER-SOL are shown in Table 4. The J_ss_ of BER-CTS-NLCs and BER-SOL were recorded as 10.63 ± 1.27 and 5.79 ± 0.79 μg cm^−2^ h^−1^, respectively. NLCs, as a type of lipid nanocarrier, have been shown to significantly increase BER penetration via paracellular and transcellular pathways [45]. Coating with a bioadhesive material, such as CTS that is capable of opening a barrier’s tight junction [46], is expected to make the NLCs even more successful in crossing barriers through the paracellular pathway.

#### 2.3.4. pH Evaluation

The pH of BER-CTS-NLCs must be determined to ensure that the system is safe to administer intranasally. The pH of nasal mucosa in its normal physiological state ranges between 4.5 to 6.5, while the pH of the BER-CTS-NLCs was observed to be 5.7. These observations suggest that the BER-CTS-NLCs formulation is physiologically compatible and expected to have no pH-related tissue-damaging effects.

### 2.4. In Vivo Experiments

#### 2.4.1. Nasal Histopathological Studies

**Figure 6:**
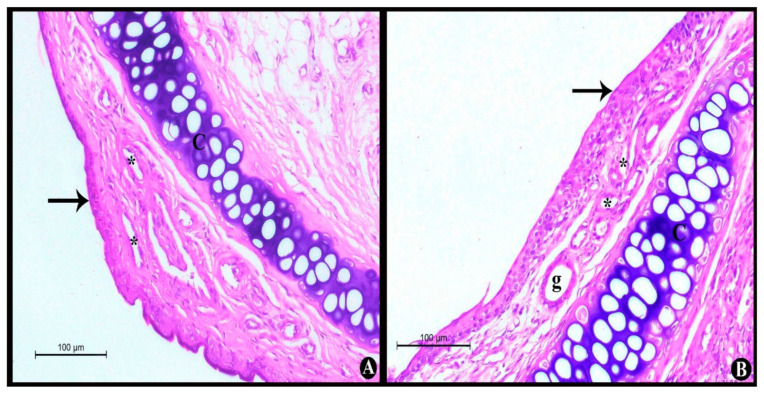
Light photomicrographs show (**A**) nasal epithelium without any administration (control); and (**B**) nasal epithelium after applying BER-CTS-NLCs. Notice normal nasal epithelium (arrows), normal lamina propria containing gland (g), capillaries (*), and normal nasal cartilage (**C**). H&E stain × 200.

A histological assessment was performed to determine the potential toxicity of the BER-CTS-NLCs system. The photomicrographs of control rats (without any administration) (Figure 6A) showed that the nasal epithelium, lamina propria, capillaries, and nasal cartilage were normal. Furthermore, histopathological analysis of nasal mucosa treated with BER-CTS-NLCs (Figure 6B) indicated findings similar to those of the control group; the nasal epithelium, lamina propria, capillaries, and nasal cartilage were normal. These results suggest that none of the excipients utilized in the BER-CTS-NLCs setup are harmful to the nasal mucosa, indicating that the BER-CTS-NLCs formulation is appropriate for nasal administration.

#### 2.4.2. Pharmacokinetics Studies and Brain Distribution of BER

**Figure 7:**
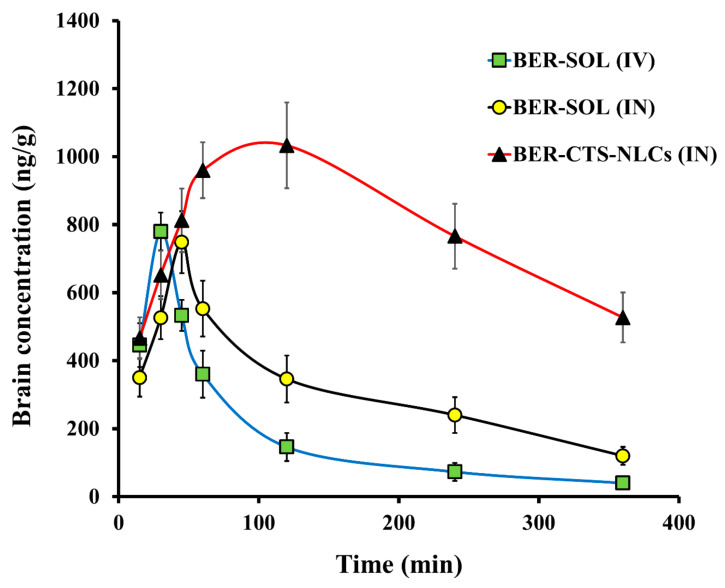
The levels of berberine (BER) in rat brain after administration of BER-SOL (IV), BER-SOL (IN), and BER-CTS-NLCs (IN).

**Figure 8:**
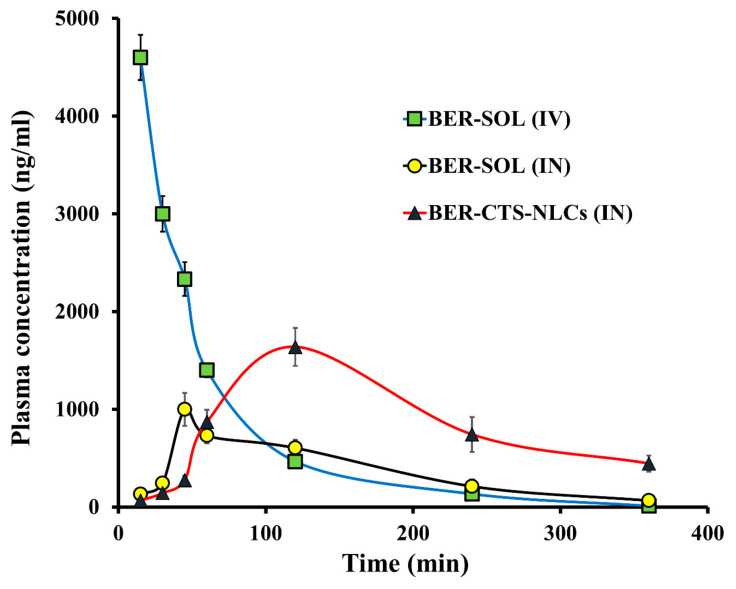
The levels of berberine (BER) in rat plasma after administration of BER-SOL (IV), BER-SOL (IN), and BER-CTS-NLCs (IN).

The pharmacokinetics and brain distribution studies were conducted using male Wistar rats after IV (BER-SOL) and IN (BER-SOL, BER-CTS-NLCs) administration. For up to 6 h, the level of BER in the brain and blood was measured (Figure 7 and Figure 8). The pharmacokinetic parameters are illustrated in Table 5. After 15 min of IV administration, the plasma concentration of BER was high (4600 ± 231.1 ng/mL) and quickly decreased, reaching 466 ± 52.4 ng/mL after 2 h. This implies that the initial high plasma levels after IV administration may be attributed to reduced BER transportation over the BBB through passive diffusion [47]. At 45 and 120 min after administration of BER-SOL (IN) and BER-CTS-NLCs (IN), peak plasma concentrations of BER were 1000 ± 168.3 and 1639 ± 194.6 ng/mL, respectively. Since the intranasal route results in systemic absorption of the medication, the presence of BER in plasma is anticipated [48]. When BER-CTS-NLCs formulation was compared to BER-SOL (IV and IN), the BER-CTS-NLCs formulation had a substantially longer (*p* < 0.05) half-life and a slower clearance rate in the brain. Between 60 and 360 min, the level of BER in the brain in the case of BER-CTS-NLCs was substantially greater (*p* < 0.05) than that in the case of BER-SOL (IV and IN). This may be because the BER-CTS-NLCs have a prolonged residence period in the nasal cavity, resulting in enhanced nasal uptake and the potential of continuously delivering the drug to the brain. The accumulation of the drug in the brain was statistically negligible within the first 45 min. This may be due to the time needed to transfer BER-CTS-NLCs and the drug’s delayed release from the lipid matrix. This has the benefit over traditional drug solutions in that the concentration of drugs in the brain may be maintained for a more extended period.

Increased BER uptake from BER-CTS-NLCs may have originated from the small PS and lipidic content of NLCs as well as from shielding against metabolic enzymes found in the nasal mucosa lining the cavity [49]. Additionally, the non-ionic surfactant poloxamer 407, utilized in the NLCs, may have enhanced drug’s absorption by increasing permeability [50].

After 360 min, the level of BER in the brain was 13.2 and 4.4 times for the NLCs formulation more than for the BER-SOL (IV) and BER-SOL (IN), respectively. These results are in line with those of Seju et al. [51]. The brain/blood ratios were determined and shown to be higher for BER-CTS-NLCs than for BER-SOL during the first 240 min (IV and IN). The brain-to-blood ratios for BER-CTS-NLCs (IN), BER-SOL (IN), and BER-SOL (IV) at 30 min were 4.56, 2.14, and 0.26, respectively. Furthermore, AUC_brain_/AUC_blood_ ratios were 1.22, 0.99, and 0.24, respectively. These results indicate direct BER transport to the brain via the intranasal route and higher accumulation of BER in the brain via the nanoparticles.

The DTP and DTE are used to determine the quantity of drugs that enter the brain directly through the olfactory pathway. These were determined utilizing data from the distribution to tissue/organs upon IV and IN administration. The highest DTE and DTP were seen in BER-CTS-NLCs, at 509.2 and 80.4, respectively, suggesting that CTS-NLCs formulations enhanced BER brain targeting compared to the solution.

NLCs may penetrate nasal epithelial cells, according to Mistry et al. [52,53]. This conclusion is evidenced by earlier studies demonstrating that nanoparticle formulations enhance brain targeting after IN administration of saquinavir, nimodipine, and risperidone [47,54,55].

## 3. Materials and Methods

### 3.1. Materials

Berberine chloride (BER), chitosan (CTS, MW: 150 kDa), poloxamer 407, glycerol monostearate (GMS) (MP: 52–54 °C and MW: 358.63), dialysis membrane (MW: 12 kDa), chloroform (HPLC grade), acetonitrile (HPLC grade), soybean lecithin (L-a phosphatidylcholine), methanol (HPLC grade), and oleic acid (OA) were procured from Sigma–Aldrich Chemical Co. (St. Louis, MO, USA). Other chemicals and reagents were of analytical grade.

### 3.2. Methods

#### 3.2.1. Design and Optimization of Experiments

NLCs were optimized in this research utilizing a Box-Behnken (BB) design. The amount of GMS (mg, X_1_), concentration of poloxamer 407 (*w*/*w*%, X_2_), and amount of OA (mg, X3) were picked as the independent variables. Each independent variable was analyzed at three levels: high, medium, and low (Table 1). The Box–Behnken design generated seventeen BER-NLCs formulations. Twelve correspond to the mid-points of each three-dimensional cube edge, and five correspond to replicas of the cube center point. The response variables were particle size (PS), entrapment efficiency (EE), zeta potential (ZP), and cumulative drug release over a 24-h period (CDR). A comparative analysis of the responses was performed using Design Expert^®^ 12.0.3.0 (Stat-Ease Inc., Minneapolis, MN, USA). Three-dimensional response surface plots were made in R using the plot3D R package [56,57]. Optimization parameters were chosen to minimize PS and maximize EE, ZP, and CDR to get the formulae with the most significant desirability factor. The selected optimal formulation was then coated with CTS.

#### 3.2.2. Preparation of Berberine Loaded NLCs (BER-NLCs)

The NLCs were fabricated using the hot homogenization and ultrasonication approach [58]. In brief, a lipid mixture (consisting of the amounts of GMS and OA identified in Table 1) was melted at 60 °C. A suitable quantity of BER (10 mg) and 0.5% lecithin (lipophilic emulsifier) were mixed with the molten lipid phase. The aqueous phase consisted of water and poloxamer 407. The aqueous phase was heated to 60 °C and then poured over the oily phase under magnetic stirring for 10 min. The dispersion was then homogenized for 6 min at 14,000 rpm. After that, the nanoemulsion was sonicated for 15 min using a probe sonicator. Finally, we allowed the formulae to cool to ambient temperature before refrigeration until further examination [59]. The components of the NLCs formulations are listed in Table 1.

#### 3.2.3. Chromatographic Conditions

BER quantity was estimated using a validated HPLC method [60]. The Agilent Eclipse-C_18_ column (4.60 mm × 25 cm, i.d., 5 μm PS) was used to detect BER quantity. A mobile phase of 0.05 mol/L NaH2PO4 and acetonitrile (70:30 *v*/*v*) (pH adjusted with phosphoric acid to 2.5) was pumped at a 1 mL/min flow rate through the HPLC system. UV detection was carried out at 345 nm at 30 °C. The injection volume and the retention time were 20 µL and 5 min, respectively. The developed HPLC assay was very sensitive and produced a linear calibration curve encompassing the concentration range 0.01–1 µg/mL (*R*^2^ = 0.999).

#### 3.2.4. BER-NLCs Characterization and Optimization

##### Particle Diameter and Surface Charge

The particle diameter and surface charge of the NLC formulations were investigated using a dynamic scattering technique (Zetasizer Nano ZS, UK) [61]. The NLC suspensions were blended with purified water (1:10) before assessment, and the analysis was performed at 25 °C [62].

##### BER Entrapment

The entrapment of BER in NLCs formulations was estimated indirectly (based on free BER). By adding 0.1N HCL to the prepared formulations, the pH was modified to 1.2 and the nanoparticles were precipitated. The formulations were centrifuged at 11,000 rpm for 45 min at 16 °C via a cooling centrifuge (SIGMA 3–30 K, Steinheim, Germany) [63]. Then, the nanoparticles were washed several times and filtrated. The quantity of BER in the filtrate was calculated using the HPLC method. The EE% was estimated as follows:

$$\mathrm{EE}\%=\frac{\left(10\;\mathrm{mg}\;-\mathrm{amount}\;\mathrm{of}\;\mathrm{BER}\;\mathrm{in}\;\mathrm{filtrate}\right)}{10\;\mathrm{mg}}\times 100$$

##### Cumulative Drug Release of BER after 24 h (CDR%)

Vertical Franz cells, with 5 cm^2^ diffusion surface area, were used to estimate the cumulative amount of BER released as a function of time. A certain amount of different BER-NLC formulations (3 mg BER in each) was added. Fifty milliliters of SNES (simulated nasal electrolyte solution) with a pH of 5.5 were poured into the receptor chamber [64]. The SNES was compounded according to a published method [65]. The receptor chamber was kept at 37 ± 0.5 °C with stirring at 50 rpm. A dialysis membrane (soaked overnight in SNES) was placed between the donor and receptor chambers. One mL of the receptor medium was collected after 24 h then filtered. The CDR% of BER in the collected samples was estimated after the analysis via the HPLC system described earlier.

#### 3.2.5. Coating of Optimized BER-NLCs with CTS (BER-CTS-NLCs)

The CTS coated BER-NLCs were formulated by dropping 5 mL of the optimized formulation to 5 mL of 0.5% CTS acetic acid solution (*w*/*v*) for 20 min under stirring at 25 °C and then refrigerating until further testing [43].

#### 3.2.6. Characterization of BER-CTS-NLCs

##### BER-CTS-NLCs Size, Surface Charge, and Morphological Evaluation

The size and surface charge of BER-CTS-NLCs were assessed, as previously stated. The morphological evaluation of BER-CTS-NLCs was evaluated using a TEM (JEOL JEM−1400, Japan) [66]. NLCs dispersions were placed onto a copper grid (300 mesh) and allowed to dry for 10 min [67]. Following drying, one drop phosphotungstic solution (1.5% *w*/*v*) was added to the copper grid and allowed to dry at 25 °C [68].

##### In Vitro Release Studies of BER

As detailed before, in vitro release experiments of BER via the dialysis membrane from BER-CTS-NLCs and BER solution (BER-SOL) were conducted using vertical Franz cells.

##### Ex Vivo Drug Permeation Studies

A Franz diffusion cell with a surface area of 2.5 cm^2^ was used to conduct ex vivo diffusion experiments. The butcher provided us with freshly removed sheep nasal mucosa, which was thoroughly cleaned to remove adhering tissues. The mucosa was then rinsed with phosphate buffer saline (PBS) and sank in PBS for 30 min [69]. A 0.2 mm thick nasal mucosa was inserted between the receptor and donor chambers. A precisely weighted volume of the optimal BER-CTS-NLCs dispersion or BER-SOL (equal to 3 mg) was placed in the donor chamber. Fifty milliliters of PBS (pH 6.5) was added to the receptor chamber. The whole setup was stirred at 50 rpm using a magnetic stirrer at 37 ± 0.5 °C. One mL was collected from the receptor chamber and replaced with 1 mL fresh PBS. The collected samples were filtered and analyzed for BER quantity using the HPLC method. The method described by Gadhave and Kokare [45] was used to calculate permeation parameters such as permeation flux and permeation coefficient.

##### Evaluation of pH

Assessment of the pH of the optimized formulation is necessary to guarantee that it does not irritate the nasal tissue. The pH was evaluated by adding 10 mL of the optimized BER-CTS-NLCs in a beaker and measuring the pH via a digital pH meter (Jenway, London, UK).

#### 3.2.7. In Vivo Experiments

The animals employed for in vivo experiments were Wistar male rats (210–245 gm). The protocol was approved by the ethics committee of the Faculty of Pharmacy at Beni-Suef University (Acceptance No: 021–211) and the Research Ethics Committee at Taif University (Ethical approval No: 43–027), which is accredited by the national committee for bioethics (No: HAO-02-T-105).

##### Nasal Histopathological Studies

Histological analysis of toxicity markers such as inflammation, abnormal symptoms, and fibrosis was used to confirm the safety of BER-CTS-NLCs intranasal administration and exclude the potential of nasal toxicity [51]. Six male Wistar rats were assigned to two classes. The first class got 30 µL of BER-CTS-NLCs (contains 5 mg/kg of BER), through intranasal administration, daily for seven days, while the second class acted as a control. After seven days, the epithelial cell membrane and nasal septum of the sacrificed rats were carefully detached and submerged in formaldehyde (10%) for 24 h, then embedded in paraffin blocks and cut into 5 mm thick pieces. Finally, the pieces were stained with hematoxylin-eosin and examined under a light microscope.

##### Pharmacokinetic Study

*Nasal Absorption and Brain Distribution Analysis of BER-CTS-NLCs.*

Wistar male rats were utilized in the pharmacokinetics study. The animals were kept in a normal laboratory environment (22 ± 3 °C, relative humidity: 30–70%). The rats were assigned to three groups: group A was given BER-SOL (IV), group B was given BER-SOL (IN), and group C was given BER-CTS-NLCs (IN). Every group was then split into six time-based subgroups of 10, 30, 60, 120, 240, and 480 min. Each subset comprised three rats; each of them got a 5 mg/kg BER dosage. In group A, BER-SOL was injected into the tail vein of rats. While in groups B and C, BER-SOL and BER-CTS-NLCs were delivered into each nostril with a micropipette attached to a low-density polyethylene tube having 0.1 mm internal diameter. Before nasal administration, the rats were anesthetized with intraperitoneal pentobarbital sodium (35–50 mg/kg). The rats were sacrificed by injecting pentobarbital sodium (overdose) at the indicated time intervals, and blood was then collected from the retino-orbital vein in EDTA coated Eppendorf tubes.

Before HPLC drug analysis, the blood was fractionated at 4000 rpm for 20 min, and the supernatant was separated and stored at −21 °C. The rats were sacrificed at the exact moment the blood was collected to remove the brain. After being cleaned twice with normal saline, the brain was weighed and cleaned of adherent tissue/fluid. A cold normal saline solution was used to homogenize the brain (normal saline: brain weight, 5:1). The brain homogenate was centrifuged at 4000 rpm (20 min, 4 °C), and then the supernatant was separated and stored at −21 °C.

##### Sample Preparation

After plasma was isolated from the blood samples, the proteins were precipitated by adding an equal volume of acetonitrile. Plasma samples were then vortexed for 3 min and then centrifuged at 11,000 rpm for 25 min. 20 μL of the supernatant was injected into the HPLC apparatus. For the brain homogenates, 100–500 µL were extracted with acetonitrile (1:1) and then vortexed for 2 min (11,000 rpm, 4 °C, 20 min); 20 μL of the supernatant was subsequently run through the HPLC system.

##### Pharmacokinetic Analysis

BER brain and plasma concentrations after IN and IV were analyzed employing the pharmacokinetic software (PK functions for Microsoft Excel, Pharsight Corporation, Mountain View, CA, USA). Additionally, metrics such as AUC, Cmax, and Tmax were computed. The profiles were utilized to directly determine the BER maximum concentration and time required to reach that concentration. The AUC was calculated up to 480 min using the linear trapezoidal method. The drug targeting efficiency (DTE), defined as the time-weighted average partitioning ratio, and the direct transport percentage (DTP), defined as the direct drug transfer from the nose to the brain, were measured as previously reported [70].

#### 3.2.8. Statistical Analysis

Each experiment was performed three times. Statistical differences between groups were evaluated where appropriate using one-way ANOVA with Tukey post hoc test, as incorporated in the aov function in R software (version 3.6.2, R Core Team, 2019). Throughout the research, a *p*-value less than 0.05 was considered significant.

## 4. Conclusions

BER-CTS-NLCs were successfully designed, optimized, and evaluated for brain targeting. All criteria determined were within acceptable ranges, including the size of particles, surface charge, and pH. BER-CTS-NLCs exhibited prolonged drug release behavior and boosted drug penetration across sheep nasal mucosa. Besides, histopathological assessment indicated that the BER-CTS-NLCs system is safe for nasal delivery. Pharmacokinetic and brain targeting studies showed that BER-CTS-NLCs (IN) had greater drug concentrations in the brain, AUC_brain_/AUC_blood_ ratio, DTP, and DTE values than BER-SOL (IN), suggesting that CTS-NLCs may be used to target the brain through the intranasal route. Lastly, according to the results of this research, BER-CTS-NLCs may be a successful approach for boosting the effect of BER in treating CNS diseases such as AD through intranasal therapy. However, substantial clinical data are required to assess the formulation’s efficacy and risk/benefit ratio in humans.

## Figures and Tables

**Table 1:** Independent and dependent variable values of NLC formulations according to Box–Behnken design.

Independent Variables			Levels				
			−1		0	1	
X_1_: GMS amount (mg)			100		150	200	
X_2_: Poloxamer 407 (% *w*/*w*)			1		1.5	2	
X_3_: Oleic acid amount (mg)			15		22.5	30	
F	X_1_ GMS (mg)	X_2_ Poloxamer 407 (*w*/*w* %)	X_3_ Oleic acid (mg)	Y_1_ PS (nm)	Y_2_ EE (%)	Y_3_ ZP (mV) ^€^	Y_4_ CDR (%)
1	−1	0	1	115.8 ± 2.4	75.2 ± 3.5	(−) 28.4 ± 2.1	84.3 ± 3.3
2	0	−1	−1	180.0 ± 3.6	67.8 ± 2.8	(−) 28.3 ± 1.5	54.2 ± 4.2
3	0	0	0	157.7 ± 3.2	78.9 ± 3.1	(−) 30.8 ± 2.4	69.8 ± 3.1
4	0	1	1	141.9 ± 2.5	81.3 ± 4.3	(−) 34.9 ± 2.3	89.6 ± 4.5
5	0	0	0	161.6 ± 3.8	81.4 ± 3.4	(−) 30.2 ± 1.6	70.3 ± 3.8
6	−1	1	0	107.2 ± 2.7	59.5 ± 3.1	(−) 29.6 ± 3.4	78.6 ± 2.1
7	1	1	0	203.2 ± 2.9	83.8 ± 3.3	(−) 29.9 ± 2.5	75.4 ± 3.5
8	0	0	0	167.0 ± 3.5	83.2 ± 2.8	(−) 34.8 ± 1.8	72.3 ± 2.4
9	0	0	0	168.4 ± 3.2	82.9 ± 4.6	(−) 30.7 ± 2.4	71.3 ± 2.9
10	−1	−1	0	132.0 ± 1.5	61.8 ± 3.6	(−) 31.5 ± 1.7	66.9 ± 3.1
11	0	−1	1	175.0 ± 4.6	85.3 ± 4.8	(−) 29.8 ± 2.4	80.5 ± 4.4
12	1	−1	0	230.8 ± 3.8	91.4 ± 3.5	(−) 31.3 ± 1.4	65.7 ± 3.2
13	1	0	1	211.6 ± 2.6	89.5 ± 2.7	(−) 35.1 ± 3.6	82.5 ± 2.3
14	−1	0	−1	125.4 ± 2.3	54.1 ± 4.6	(−) 33.1 ± 2.7	59.3 ± 2.8
15	1	0	−1	218.7 ± 5.2	86.9 ± 2.4	(−) 27.5 ± 1.9	57.3 ± 3.1
16	0	1	−1	148.0 ± 3.1	65.4 ± 6.2	(−) 32.1 ± 1.3	61.4 ± 4.2
17	0	0	0	171.3 ± 2.5	84.3 ± 4.9	(−) 28.9 ± 2.3	71.8 ± 5.2

^€^ Excluded from optimization due to insignificance.

**Table 2:** The design expert results of all response variables.

Source	Size (nm)		EE%		CDR%	
	F	*p*-Value	F	*p*-Value		
Model	416.50	<0.0001	162.22	<0.0001	505.75	<0.0001
X_1_: GMS amount (mg)	1134.32	<0.0001	670.22	<0.0001	8.19	0.0133
X_2_: Poloxamer 407 (% *w*/*w*)	108.84	<0.0001	13.59	0.0050	173.20	<0.0001
X_3_: Oleic acid amount (mg)	6.34	0.0257	241.61	<0.0001	1335.85	<0.0001
X_1×3_			85.47	<0.0001		
X_1_^2^			54.30	<0.0001		
X_2_^2^			43.39	0.0001		
X_3_^2^			14.84	0.0039		
Lack of Fit	0.3386	0.9183	0.4527	0.7962	0.9338	0.5748
Model	Linear		Reduced Quadratic		Linear	
SD	0.1573		0.0003		1.01	
R^2^	0.9897		0.9921		0.9915	
Adequate precision	64.3041		42.7075		72.4640	
Predicted R^2^	0.9845		0.9687		0.9849	
Adjusted R^2^	0.9873		0.9860		0.9895	
%CV	1.23		1.98		1.42	
Sqrt (PS) = 12.7979 + 1.87247 × X_1_ − 0.580015 × X_2_ − 0.140025 × X_3_. 1/(EE) = 0.0121807 − 0.0024 × X_1_ + 0.0003 × X_2_ − 0.0014 × X_3_ + 0.0012 × X_1_ .X_3_ + 0.0009 × X_1_^2^ + 0.0008 × X_2_^2^ + 0.0005 × X_3_^2^. CDR% = 71.2471 − 1.025 × X_1_ + 4.7125 × X_2_ + 13.0875 × X_3_.						

**Table 3:** Composition, experimental, and Design Expert^®^ predicted values of the optimal BER-CTS-NLCs formulation.

Independent Factors	Optimal Value	Response	ExperimentalValue	Design Expert^®^ Predicted Value	Prediction Error (%) *
X_1_: GMS amount (mg)	114.7	PS (nm)	142.1 ± 5.7	119.8	15.7
X_2_: Poloxamer 407 (% *w*/*w*)	1.8	EE%	80.3 ± 3.1	76.1	5.2
X_3_: Oleic acid amount (mg)	30	CDR%	85.6 ± 4.2	88.2	3

* Calculated as (Experimental value-Design Expert^®^ predicted value)/Experimental value × 100.

**Table 4:** Ex vivo permeation parameters of BER-CTS-NLCs vs. BER-SOL.

Preparation	Flux Jss (µg cm^−2^ h^−1^)	Cumulative BER Permeated at 12 h (μg/cm^2^)	Permeability Coefficient (cm/h)
BER-CTS-NLCs	10.63 ± 1.27	472.8 ± 25.87	0.01063 ± 0.00027
BER-SOL	5.79 ± 0.79	271.2 ± 19.25	0.00579 ± 0.00036

**Table 5:** Pharmacokinetic parameters of BER-SOL (IV), BER-SOL (IN), and BER-CTS-NLCs (IN).

Formulation	Tissue/Organ	T_max_ (min)	C_max_ (ng/mL)	Ke (min^−1^)	t_1/2_ (min)	AUC_0–t_ (ng/mL × min)	C_brain_/C_blood_ at 30 min.	AUC_brain_/AUC_blood_
BER-SOL (IV)	Brain	30	780	0.005	128.5	74,944	0.26	0.24
	Blood	15	4600	0.017	41.5	313,493		
BER-SOL (IN)	Brain	45	748	0.005	144.6	137,275	2.14	0.99
	Blood	45	1000	0.009	75.1	139,171		
BER-CTS-NLCs (IN)	Brain	120	1033	0.003	247.2	469,403	4.56	1.22
	Blood	120	1639	0.005	127.8	385,609		

## Data Availability

Data sharing contains in this article.
